# Dramatic long-term restoration of an oak woodland due to multiple, sustained management treatments

**DOI:** 10.1371/journal.pone.0241061

**Published:** 2020-10-23

**Authors:** Karen Glennemeier, Stephen Packard, Greg Spyreas

**Affiliations:** 1 Habitat Research LLC, Wilmette, Illinois, United States of America; 2 North Branch Restoration Project, Northbrook, Illinois, United States of America; 3 Illinois Natural History Survey, University of Illinois, Champaign, Illinois, United States of America; Technical University in Zvolen, SLOVAKIA

## Abstract

We measured 34 years of plant community change in a degraded oak woodland undergoing ecological management. Management included regular prescribed fire, control of white-tailed deer populations, repeated sowing of a diverse seed mix, and removal of invasive plants. We tracked change with several conservation metrics. Time series analysis showed no significant changes over time in either plant species richness or the Shannon-Weiner diversity index. Floristic Quality Assessment measures—the Floristic Quality Index (FQI), Cover-weighted FQI, and the Mean Coefficient of Conservatism (Mean *C*)—all increased dramatically over time, such that their values now surpass those of the highest quality representative of this habitat in the region. Cover-weighted FQI had the added benefit of being quick to respond (negatively and positively) to short-term management changes during the study. This sensitivity highlights its utility for adaptive management, enabling timely, data-driven changes to ongoing management regimes. Plant community composition showed striking changes during the study period, as species of high conservation value replaced weedier species. As a group, conservative woodland species are notoriously slow to recover from degradation, making this flora’s recovery particularly notable. A mid-study cessation of management immediately stalled the woodland’s recovery according to Floristic Quality metrics, but the restoration quickly returned to its positive trajectory with the resumption of management treatments. These results illustrate that impressive plant biodiversity restoration can be achieved, even in highly degraded contemporary oak ecosystems, if ecological management is comprehensive and if it is sustained over time.

## Introduction

Oak woodlands often show little or no recovery over time after they have been degraded [1–3]. This is typically due **to** lack of fire, excess shade from fire-intolerant species (“mesophication”), invasive species, deer overpopulation, habitat fragmentation, and/or a lack of native plant propagules [4–6]. Ecological management is widely agreed to be necessary to overcome these barriers to the restoration of woodland health and diversity. However, in modern landscapes, unpredictable and disappointing outcomes often characterize both actively restored natural areas and those undergoing passive succession [7,8]. Even very expensive vegetation restoration projects fail to meet their conservation goals more often than not (e.g., as measured by plant conservation and biodiversity metrics; [9,10]). Therefore, if confidence in the methods used for natural areas management and restoration is to progress, and if needed resources are to be justified, it is crucial to rigorously demonstrate need and efficacy.

To this point, most studies have examined specific individual management techniques (e.g., fire [11]; brush removal [12]), despite the fact that few land managers employ only a single management technique at a given site. Because ecological processes, stressors, or management actions may interact synergistically or antagonistically in combination [13,14], studies examining multiple ecological management treatments simultaneously may be especially useful for land managers. In addition, long-term studies of management impacts are rare (but see [15–17]). Given limited time and resources, most restorations undergoing ecological management stop monitoring vegetation after 3–6 years and rarely extend past ten years (e.g., wetland mitigation projects, [18–20]). However, changes in plant communities can be highly irregular over time, and habitats often quickly regress after initial, short-term improvement. This instability is especially common in young restorations and/or where exotic species take hold [9,21–23]. Therefore, long-term, multi-technique studies of management/restoration regimes are needed to illustrate where and why lasting restoration success occurs and to provide the most actionable information to land managers.

Tracking management outcomes requires effective indices that can convey both short-term changes and long-term trends in vegetation. Many indices, with varying degrees of complexity, have been developed to describe the biodiversity and integrity of biological communities, and opinions vary as to which are the most useful (see [9,24–27]). Plant diversity measures via a simple species count, or evenness measures such as the Shannon index, are two of the most commonly used (e.g., [28,29]) and easily understood [30]. Other indices have been developed to more directly assess changes in plant community composition. For example, Swink and Wilhelm [31] introduced Floristic Quality Assessment (FQA) to provide a means of distinguishing differences among plant communities, based on the degree to which their component species are reflective of high-conservation value, native remnant habitats that are relatively free of anthropogenic disturbance. “Floristic Quality” is measured via two principal metrics: the Floristic Quality Index (FQI) and the Mean *C* (for further description see “Indices” below). These have been widely used to distinguish habitat quality, conservation value, management history, successional status, and restoration success among habitat types (e.g., [12,26,32–35]). They also have been used to describe changes within plant communities over time (e.g., [8,23,36]).

The objectives of this study were as follows:

1. To learn how a degraded oak woodland’s plant community changed under a long-term, multi-treatment, ecological management regime.
2. To investigate which of five common metrics would best quantify management success/failure and restoration progress over the life of the study, as measured by the plant community’s diversity and conservation value.
3. To learn how an abrupt, mid-study cessation of the management treatments affected the woodland recovery.
4. To evaluate the ability of the five metrics to reflect this mid-study disruption in management, as quickly detecting and adjusting to change is the key to successful restoration work (i.e., adaptive management).

To address these four objectives, we analyzed a 34-year vegetation data set from an oak woodland in northeastern Illinois that received the following treatments: regular prescribed fire, invasive species removal/control, brush cutting, stand thinning, deer population management, and seeding of native plants.

## Methods

### The study area

Vestal Grove is an oak woodland that is part of the 33 ha (83 acre) Somme Prairie Grove forest preserve in Northbrook, Illinois (centroid 42° 8’20.00"N, 87°49’45.00"W), which is part of the Forest Preserves of Cook County (FPCC)’s 69,000-acre preserve system. Vestal Grove measures approximately 200 meters east to west and ranges from 75 to 200 meters north to south, on the west-facing slope of the Deerfield Lobe of the Lake Border Moraine. Soils are Markham silt loam (2 to 5 percent slopes) and Beecher Silt Loam (U.S.D.A. 1979). It is a relatively isolated suburban preserve that is surrounded by roads, railroads, and developed land.

According to the Public Land Survey of 1839, the indigenous landscape contained widely scattered oaks, characteristic of the region’s oak woodlands or savanna [37]. Aerial photographs taken in 1938 showed the surrounding area to be crop fields and pasture, with the study area being a wooded pasture. When restoration began in 1983, Vestal Grove contained a dense understory of the invasive exotic *Rhamnus cathartica* (common buckthorn), with a depauperate spring flora and little herbaceous vegetation of any kind in summer or fall. However, nearby brushy edges and old-field pasture vegetation contained many species characteristic of savannas and oak woodlands. Mature trees were mostly *Quercus macrocarpa* (bur oak), with lesser numbers of *Q*. *elipsoidalis* (Hill’s oak) and *Carya ovata* (shagbark hickory). Such woodlands would have incurred frequent fire before widespread European settlement and industrialization [38].

### Ecological management

#### Fire

We initiated restoration efforts with dormant season burns in 1983 and 1984. We burned in spring or fall every second year, on average, through the present, with the exception of the moratorium years.

#### Seeding

In fall 1985, we began the first of several annual seed broadcasts in an attempt to restore a native vegetation matrix. Seed mixes included 129 species characteristic of open oak woodlands [31,39] and were collected from spontaneous populations within 25 miles of the study site (S1 Table). After the end of the moratorium, we again broadcast seed over the entire area annually for two years. In recent years, seed broadcasts have been occasional and focused in the small areas where the canopy had recently been thinned by pole tree removal. Not all species were seeded in every broadcast, with the largest numbers concentrated in the initial two years and the two post-moratorium years. Some seeds were more available for collection in some years than others, and the supplemental broadcasts were limited to the species deemed most appropriate for these smaller target areas.

#### Weed control, brush cutting, and stand thinning

Plant removal or control had two goals: 1) to remove invasive (largely non-native) species, and 2) to open up the area’s oak woodland canopy to better reflect the needs of more light-demanding woodland/savanna species. We largely eliminated a patchy but dense population of *Alliaria petiolata* (garlic mustard) by pulling. We initially scythed *Cirsium arvense* (creeping thistle), *Rubus* spp. (briars), and *Solidago altissima* (tall goldenrod), but they seemed to decrease over time on their own, so we did not target them thereafter. It should be noted that of the 10 non-native species in the initial sample area (S2 Table), we made no effort to control or directly remove seven of them at any time (we controlled only *Rhamnus cathartica*, *Lonicera tatarica*, and *Cirsium vulgare*). For the first 13 years, we controlled invasive brush such as *R*. *cathartica* by burning alone, which top-killed the stems. Most of them re-sprouted between burns, with multiple small stems per plant. During the management moratorium (see below), these re-sprouts grew to dense stands, 1- to 2.5-meters tall. In 2003, at the end of the moratorium, we cut all large invasive stems, treated the stumps with 20% Garlon, and foliar-sprayed re-sprouts with 3–5% Garlon. In recent years, we have cut maturing trees (under ≈50 cm [20 inch] diameter-at-breast height) to increase understory light to levels that would likely support bur oak reproduction [40]. This included cutting all non-native, and some native trees.

#### Deer control

While regional white-tailed deer population numbers have been extraordinarily high for some time [41], unsystematic deer counts and site stewards’ observations of unusually heavy browse on even less-preferred plant species provided anecdotal indications that deer numbers at Somme Prairie Grove increased dramatically between 1991–93 (personal communication). In accordance, FPCC census counts estimated deer numbers in the area had risen to more than 62 deer per km^2^ by 1991 (unpublished data). Because of excessive deer-automobile collisions and because the ecosystem is believed to sustainably support only 3 to 6 deer per km^2^ (e.g., pre-European settlement [42,43]), both FPCC and the surrounding Village of Northbrook began deer control programs to reduce their numbers on site in 1993. By 1994, these programs had brought the numbers down substantially (unpublished FPCC data). Deer culling has continued since then.

#### The management moratorium

In the fall of 1996, a restoration “moratorium” was instated by the FPCC board president, and all vegetation management techniques (described above) were halted on all sites in the District in response to a campaign of protests against controlled burns, use of herbicide, and cutting trees. The moratorium was gradually lifted, but the Vestal Grove transect area went without management from September 1996 until July 2003. Staff and volunteers resumed restoration activities with a major brush cut on 12 July 2003 and a burn on 9 November 2004. Regular management has continued since this time, as described above. (For more on the management of this site, see [44]).

### Plant sampling

Fifteen circular, 1-m^2^ permanent plots were established in 1986, beginning at a random location and continuing at 10-m intervals along a 150-m linear transect through Vestal Grove. The transect was marked by metal posts placed at 50-m intervals. Plots were placed one meter north of the transect. Species name and direct estimates of percent aerial cover for all herbaceous plants were recorded, as well as of those woody plants with foliage below one meter tall. In order to represent both early and later season flora, plots were sampled twice–in late spring and late summer. To compile a single sample, the species lists from the two plot samples were combined and, if a species was sampled at both times, the larger cover value was used. The transect was sampled roughly every two years.

The first sampling was done in fall 1985 and spring 1986, and the two were combined to constitute the 85/86 sample. Although the first seeding was done in fall 1985, we considered the initial sampling to provide a good representation of pre-seeding vegetation, as the seeded species would not have emerged sufficiently in 1986 to be recognized or counted in spring samples. The fall 1985 data come from a different arbitrary transect that had been established separate from the current study. This transect was very near (4.1 meters from) and parallel to the 1986 permanent transect. Previous studies confirm that years effects are generally negligible for our chosen indices [45].

### Indices

We compared change over time in the values of several biodiversity and conservation indices commonly used to reflect ecosystem health or biological degradation. We calculated these indices for all species as well as for native species only. Species richness was a simple species count, represented as a per-plot mean. Mean *C* was the average Coefficient of Conservatism (*C*) for the species within the plots, taken as a per-plot mean. Each plant in the Chicago region has previously been assigned a *C*-value by collective, expert judgment to indicate the degree to which the species is faithful to high-quality, remnant, natural communities [31]. *C*-values are integers that range from 0 to 10. The species virtually restricted to high-quality natural areas have *C*-values of 9 or 10, whereas species that may also occur in habitats that have been badly damaged by anthropogenic disturbance have been given *C*-values of 0 or 1.

The Floristic Quality Index, or FQI, was calculated using the number of species and the Mean *C* [31]. Specifically, FQI = (Mean *C*) x √N, where C is the Coefficient of Conservatism and N is the number of species.

For the mean Cover-weighted FQI per plot, we first calculated Cover-weighted Mean *C* as follows: $\frac{\sum_{i=1}^{S}c_{i}p_{i}}{\sum_{i=1}^{S}p_{i}}$, where *c* is the C-value of species *i*, and *p* is the percent cover of species *i* within the plot, for all *S* species within the plot. We then used this Cover-weighted Mean *C* in the calculation of FQI, as above.

The mean Shannon-Weiner Index (H’) per plot measured the degree to which each plot was dominated by one or a few species and was calculated as follows: H’ = —Σp_i_ ln p_i_, where p_i_ is the proportion of individuals of the species i within the plot [30].

Finally, to examine changes in composition over time in greater detail, species were grouped into four “conservatism classes.” Species data were grouped into the following classes: A-0, 1–3, 4–6, and 7–10, with “A” indicating adventive, or non-native, species. Percent cover of species within each class was summed for each plot and then represented as a per-plot mean.

### Statistical analysis

We applied statistical tests to the indices incorporating all species, but not to those using only native species. (In the relevant figures, we have provided the trends for indices using native species alongside those for all species to illustrate the similar patterns shown by each.) To examine the hypothesis that each index changed predictably over time (i.e., there was a statistically significant positive or negative trend over time), we conducted time series analyses using the augmented Dickey-Fuller test [46]. Our method removes the autocorrelation inherent in re-sampled time series data and does not pre-suppose any particular shape of the hypothesized trend lines. To model changes in species metrics, we compared a pure random walk (or random “noise”) model with those that added an intercept (or drift) term, a lagged index term, and a linear time trend term. Any drift, lag, or linear term would indicate predictable dependence of the current year’s value on that of the prior year, rather than simply random noise. The best model fit was considered the most parsimonious model that exhibited both highly significant test statistics (*P* < 0.01) and a robust adjusted R^2^ value. Analysis was conducted using the R open-source software program [47].

To test the significance of changes in species composition, we used repeated measures multivariate analysis of variance (MANOVA) to compare mean cover within four conservatism classes in the first and most recent years of the study (years 1986 and 2019), followed by ANOVAs for each conservatism class. Analysis was conducted using the SPSS software package [48]. We supplemented statistical analyses with qualitative examinations of species composition patterns over time, displayed graphically in figures.

## Results

**Fig 1:**
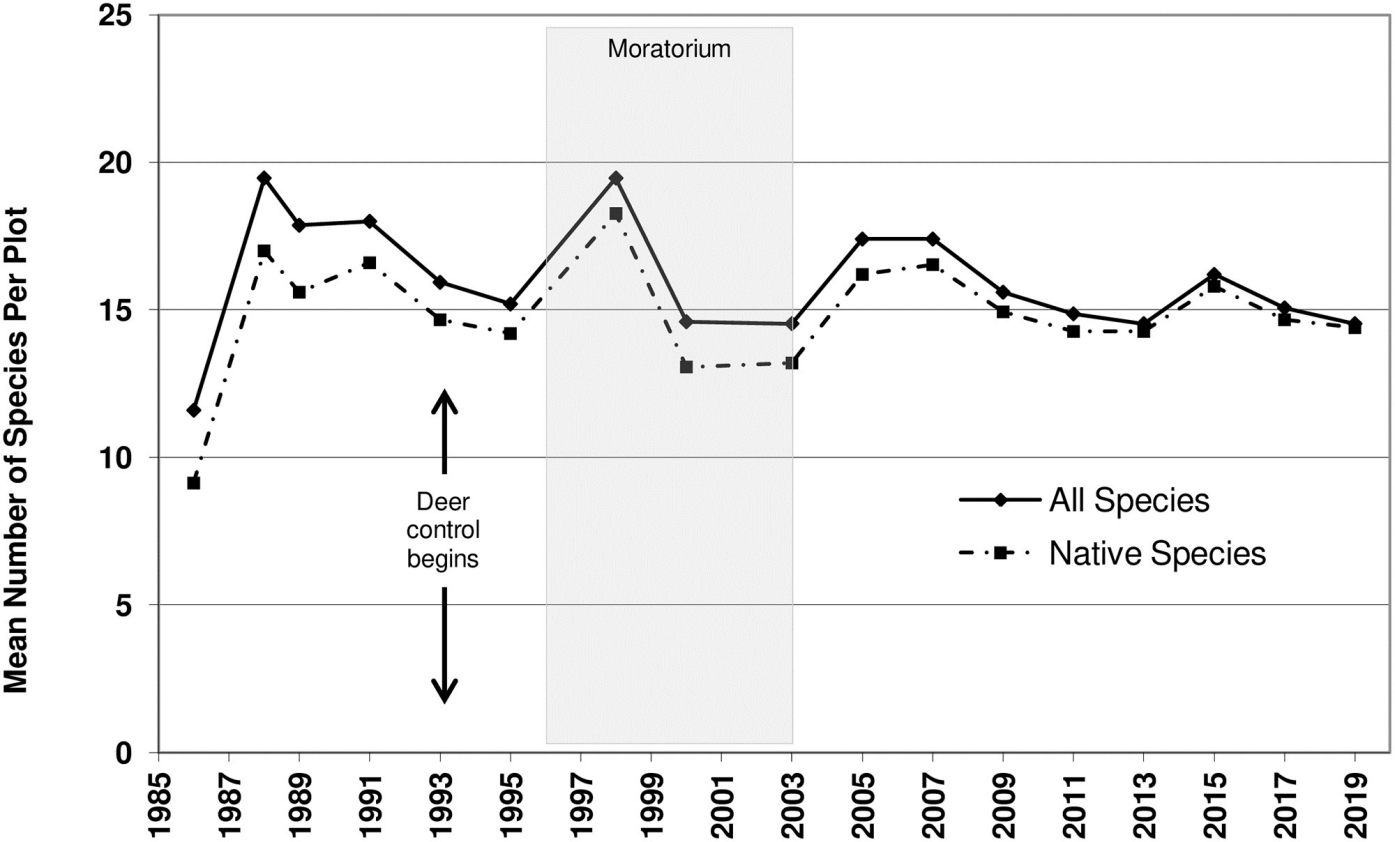
Species richness, represented as the mean number of species per plot. Time series analysis used the mean value of all species for each year sampled. Arrows represent the onset of deer culling, which continued thereafter. Shaded area represents the vegetation management moratorium years.

**Fig 2:**
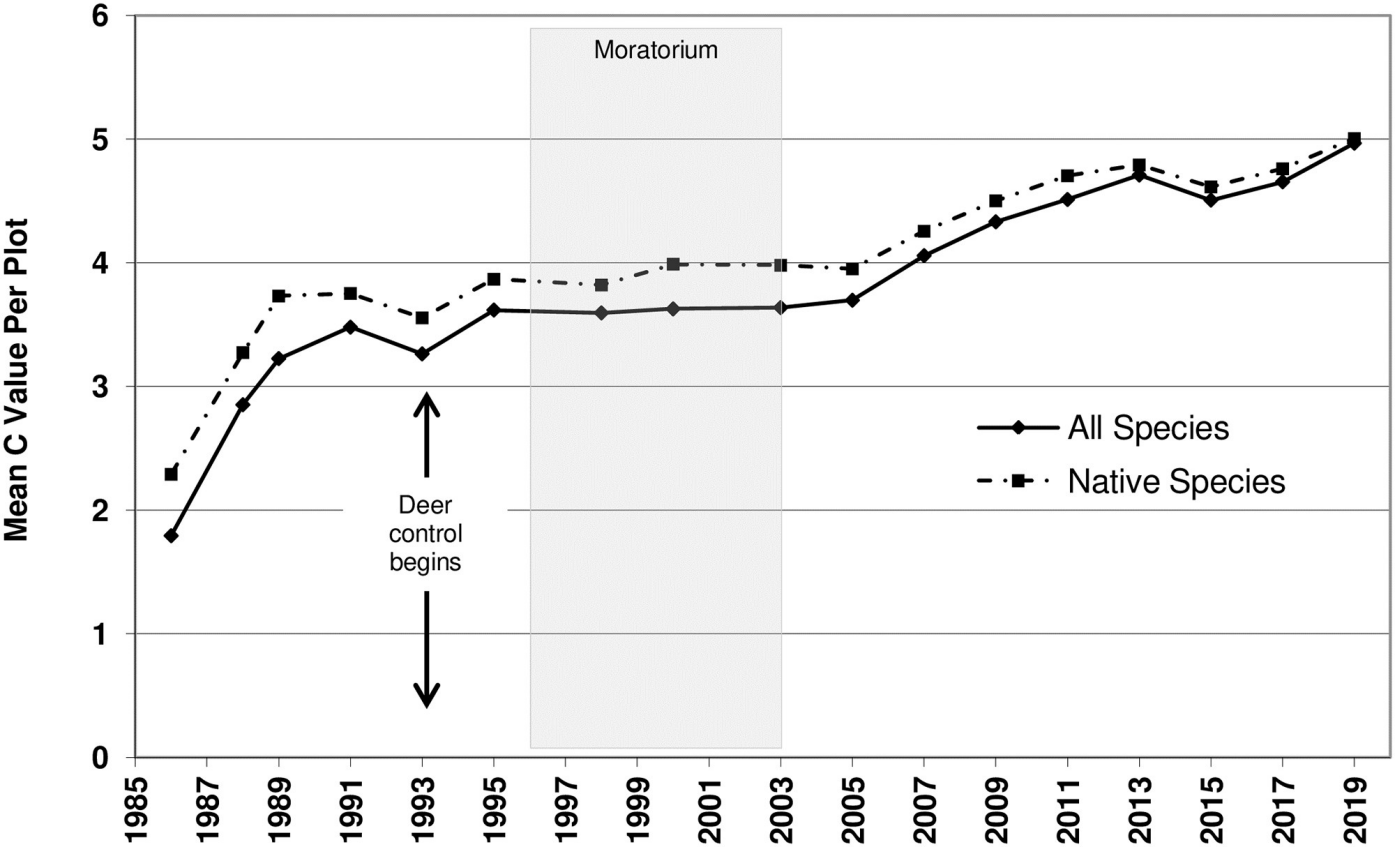
Mean *C*-Value per plot. Time series analysis used the mean value of all species for each year sampled. Arrows represent the onset of deer culling, which continued thereafter. Shaded area represents the vegetation management moratorium years.

**Fig 3:**
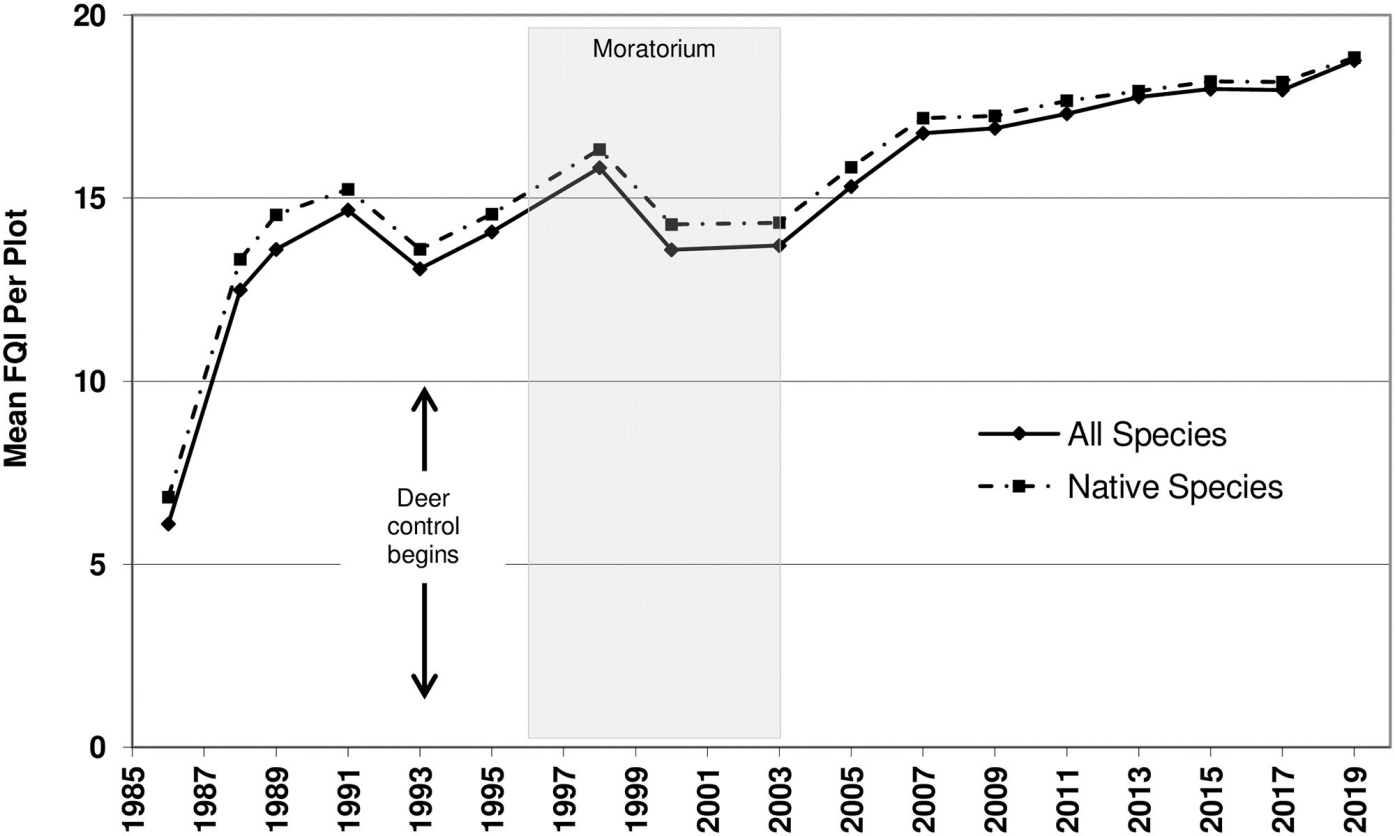
Mean Floristic Quality Index (FQI) per plot. Time series analysis used the mean value of all species for each year sampled. Arrows represent the onset of deer culling, which continued thereafter. Shaded area represents the vegetation management moratorium years.

**Fig 4:**
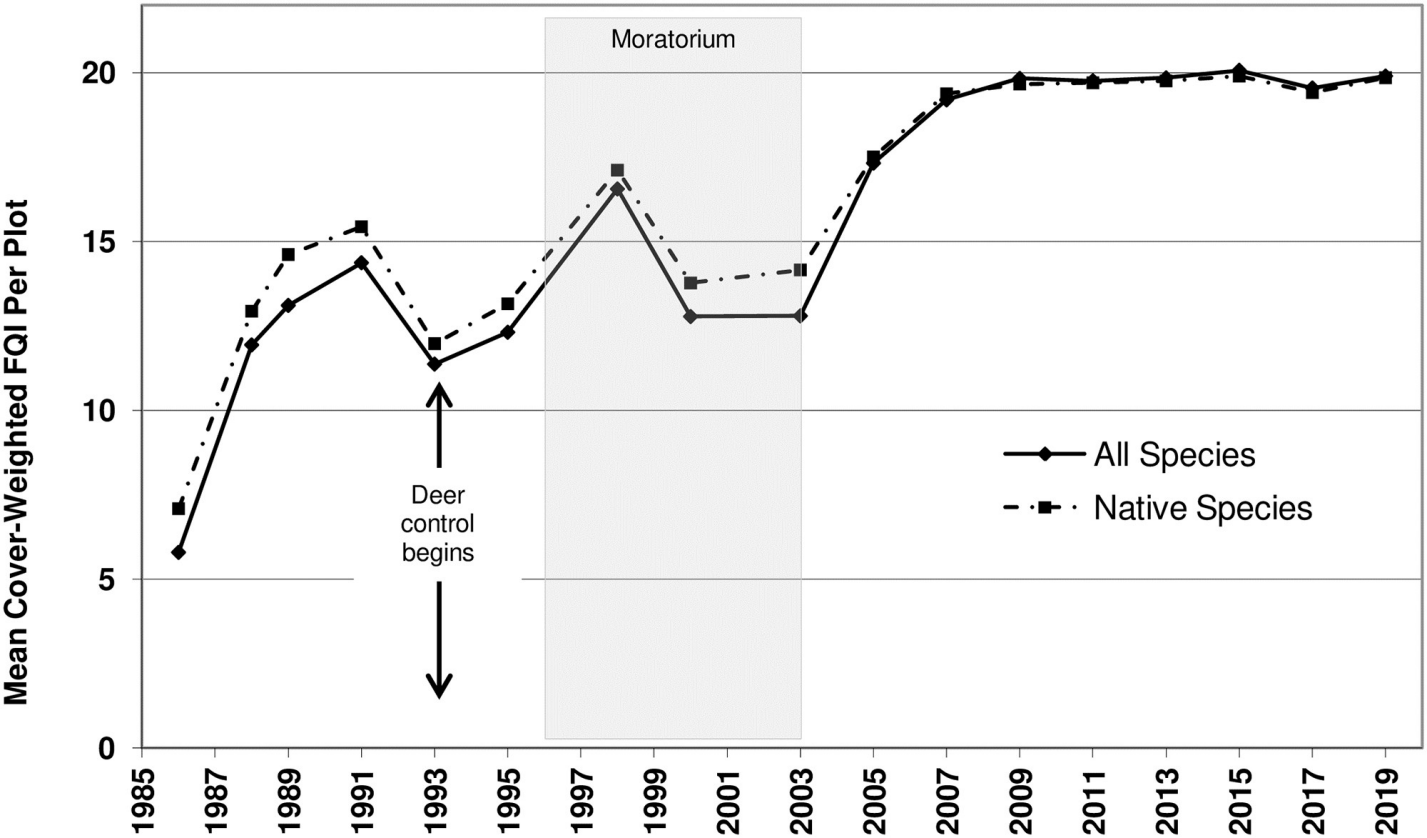
Mean Cover-weighted Floristic Quality Index (FQI) per plot. Time series analysis used the mean value of all species for each year sampled. Arrows represent the onset of deer culling, which continued thereafter. Shaded area represents the vegetation management moratorium years.

**Fig 5:**
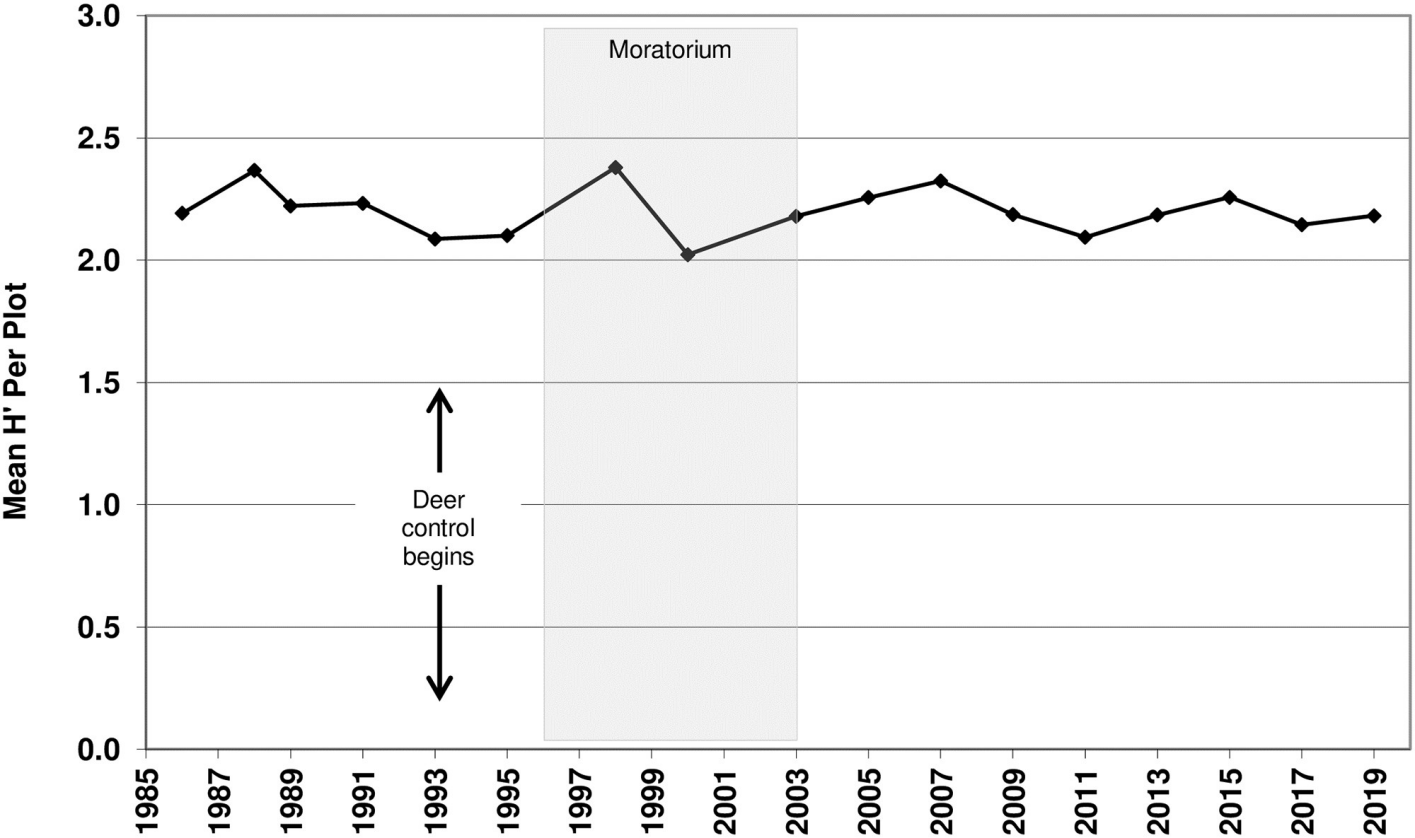
Mean Shannon-Weiner Diversity Index (H’) per plot. Time series analysis used the mean value for each year sampled. Arrows represent the onset of deer culling, which continued thereafter. Shaded area represents the vegetation management moratorium years.

Time series analysis showed no significant changes over time in either plant species richness or the Shannon-Weiner diversity index, whereas Floristic Quality Assessment measures, including the Floristic Quality Index (FQI), Cover-weighted FQI, and the Mean Coefficient of Conservatism (Mean *C*), all increased dramatically over time (Figs 1–5). Although statistical significance values for Cover-weighted FQI suggested a weak overall trend, visual examination of the trend confirms that the actual relationship was strong (see below and in “Discussion”).

Table 1 presents the model output for each of the five indices calculated, including an indication of the best model fit for each index. Table 2 provides critical values for use in comparing models. As shown in Table 1, for none of the indices was the random walk (null) model the best fit, suggesting that each index could be modeled with a predictable pattern, rather than merely representing “noise.”

**Table 1:** Augmented Dickey-Fuller tests.

**Species Richness**				
**Model equation (Best model fit in bold)**	**Estimates**	***P*-values**	**Adj R**	**Test statistics**
Δ*y*_*t*_ = γ*y*_*t-1*_ + ε_*t*_	γ = -3.46e-03	γ: *p* = 0.934	-0.0662	τ_1_ = -0.0840
**Δ*y***_***t***_ **= *a***_**0**_ **+ γ*y***_***t-1***_ **+ ε**_***t***_	*a*_0_ = 18.2	*a*_0_: *p* = 1.23e-04**	0.637	τ_2_ = -5.23**
	γ = -1.14	γ: *p* = 1.27e-04**		φ_1_ = 13.8**
Δ*y*_*t*_ = *a*_0_ + γ*y*_*t-1*_ + *a*_*2*_*t* + ε_*t*_	*a*_0_ = 19.7	*a*_0_: *p* = 4.14e-05**	0.697	τ_3_ = -5.79**
	γ = -1.15	γ: *p* = 6.25e-05**		φ_2_ = 12.2**
	*a*_*2*_ = -0.152	*a*_*2*_: *p* = 0.0741		φ_3_ = 18.3**
**Mean *C***				
**Model equation (Best model fit in bold)**	**Estimates**	***P*-values**	**Adj R**	**Test statistics**
Δ*y*_*t*_ = γ*y*_*t-1*_ + ε_*t*_	γ = 0.0407	γ: *p* = 0.128	0.090	τ_1_ = 1.61
Δ*y*_*t*_ = *a*_0_ + γ*y*_*t-1*_ + ε_*t*_	*a*_0_ = 1.40	*a*_0_: *p* = 3.57e-03**	0.356	τ_2_ = -3.05*
	γ = -0.319	γ: *p* = 8.69e-03**		φ_1_ = 8.37**
**Δ*y***_***t***_ **= *a***_**0**_ **+ γ*y***_***t-1***_ **+ *a***_***2***_***t* + ε**_***t***_	*a*_0_ = 2.82	*a*_0_: *p* = 8.66e-06**	0.737	τ_3_ = -6.27**
	γ = -0.929	γ: *p* = 2.86e-05**		φ_2_ = 20.8**
	*a*_*2*_ = 0.104	*a*_*2*_: *p* = 4.83e-04**		φ_3_ = 22.0**
**FQI**				
**Model equation (Best model fit in bold)**	**Estimates**	***P*-values**	**Adj R**	**Test statistics**
Δ*y*_*t*_ = γ*y*_*t-1*_ + ε_*t*_	γ = 0.0327	γ: *p* = 0.375	-0.0103	τ_1_ = 0.915
Δ*y*_*t*_ = *a*_0_ + γ*y*_*t-1*_ + ε_*t*_	*a*_0_ = 8.78	*a*_0_: *p* = 3.74e-04**	0.539	τ_2_ = -4.31**
	γ = -0.535	γ: *p* = 7.24e-04**		φ_1_ = 11.8**
**Δ*y***_***t***_ **= *a***_**0**_ **+ γ*y***_***t-1***_ **+ *a***_***2***_***t* + ε**_***t***_	*a*_0_ = 13.1	*a*_0_: *p* = 1.12e-06**	0.809	τ_3_ = -7.62**
	γ = -1.04	γ: *p* = 3.78e-06**		φ_2_ = 26.0**
	*a*_*2*_ = 0.383	*a*_*2*_: *p* = 5.29e-04**		φ_3_ = 32.9**
**Cover-weighted FQI**				
**Model equation (None was a strong fit)**	**Estimates**	***P*-values**	**Adj R**	**Test statistics**
Δ*y*_*t*_ = γ*y*_*t-1*_ + ε_*t*_	γ = 0.0294	γ: *p* = 0.515	-0.0360	τ_1_ = 0.667
Δ*y*_*t*_ = *a*_0_ + γ*y*_*t-1*_ + ε_*t*_	*a*_0_ = 6.61	*a*_0_: *p* = 0.0111*	0.281	τ_2_ = -2.62
	γ = -0.371	γ: *p* = 0.0201*		φ_1_ = 4.61
Δ*y*_*t*_ = *a*_0_ + γ*y*_*t-1*_ + *a*_*2*_*t* + ε_*t*_	*a*_0_ = 10.5	*a*_0_: *p* = 5.56e-04**	0.518	τ_3_ = -4.02*
	γ = -0.94	γ: *p* = 1.46e-03**		φ_2_ = 7.20*
	*a*_*2*_ = 0.579	*a*_*2*_: *p* = 0.0149*		φ_3_ = 9.05*
**Mean H’ (Shannon-Weiner Index)**				
**Model equation (Best model fit in bold)**	**Estimates**	***P*-values**	**Adj R**	**Test statistics**
Δ*y*_*t*_ = γ*y*_*t-1*_ + ε_*t*_	γ = -2.61e-03	γ: *p* = 0.891	-0.0653	τ_1_ = -0.140
**Δ*y***_***t***_ **= *a***_**0**_ **+ γ*y***_***t-1***_ **+ ε**_***t***_	*a*_0_ = 2.78	*a*_0_: *p* = 2.22e-04**	0.609	τ_2_ = -4.93**
	γ = -1.27	γ: *p* = 2.20e-04**		φ_1_ = 12.2**
Δ*y*_*t*_ = *a*_0_ + γ*y*_*t-1*_ + *a*_*2*_*t* + ε_*t*_	*a*_0_ = 2.78	*a*_0_: *p* = 3.38e-04**	0.589	τ_3_ = -4.85**
	γ = -1.28	*a*_0_: *p* = 3.38e-04**		φ_2_ = 7.83*
	*a*_*2*_ = 3.23e-03	*a*_*2*_: *p* = 0.581		φ_3_ = 11.7**

We present three different regression equations for each index, to test the null hypothesis of an unpredictable pattern in change over time, using augmented Dickey-Fuller tests as in [46]. The first equation represents a pure random walk (“noise”) model. The second adds an intercept, or drift, term (*a*_0_). The third includes both a drift and a linear time trend (*a*_*2*_). The parameter of interest for all three is γ, which is tested with the τ test statistic. For the first equation, if we cannot reject the null that γ = 0, then the change in y would simply represent random noise. For the second and third equations, γ = 0 would mean there is a random noise component, but we don’t know if the intercept and time trend are zero. To test the intercept and time components, the test statistic is φ, which looks at the joint hypotheses that *a*_0_ = γ = *a*_*2*_ = 0, or various subsets of these parameters. Critical values for τ and φ can be found in Table 2.

* Statistically significant (*p* < 5%).

** Statistically significant (*p* < 1%).

**Table 2:** Critical values for each Dickey-Fuller test statistic.

Test statistic	1%	5%	10%
τ_1_	-2.66	-1.95	-1.60
τ_2_	-3.75	-3.00	-2.63
φ_1_	7.88	5.18	4.12
τ_3_	-4.38	-3.60	-3.24
φ_2_	8.21	5.68	4.67
φ_3_	10.61	7.24	5.91

Critical values are based on the empirical cumulative distribution of τ and the empirical distribution of φ, for N ≤ 25 (as reprinted in [46]; original source [49]).

For species richness, the best model included a lag term, which indicates that species richness changed according to a predictable pattern based on the prior year’s value. Adding a time component to the model did not appreciably improve the model fit, suggesting that species richness was neither increasing nor decreasing over time, but instead remained steady at around 15 species per plot (Fig 1).

For both Mean *C* and FQI, the addition of a time component substantially improved the model fit, illustrating a statistically significant and increasing trend over time, with per-plot Mean *C* increasing from a value of 2 to 5 and FQI going from 6 to 19 (Figs 2 and 3).

Cover-weighted FQI rose from 6 to 20 and was the only metric whose mid-survey dip appeared to affect its overall statistical significance (Fig 4). In other words, it appears that an exogenous factor—most probably these mid-survey dips—overrode the ability of time alone to explain the trend, as evidenced by weaker significance values compared to the non-weighted FQI model, despite similar magnitudes in the two metrics’ overall, upward trends.

For Mean H’ (Shannon-Weiner Index), the best model included a lag term, indicating that Mean H’ changed according to a predictable pattern based on the prior year’s value. Adding a time component to the model did not appreciably improve the model fit, suggesting that Mean H’ was neither increasing nor decreasing with time but instead remained steady at around 2.2 (Fig 5).

**Fig 6:**
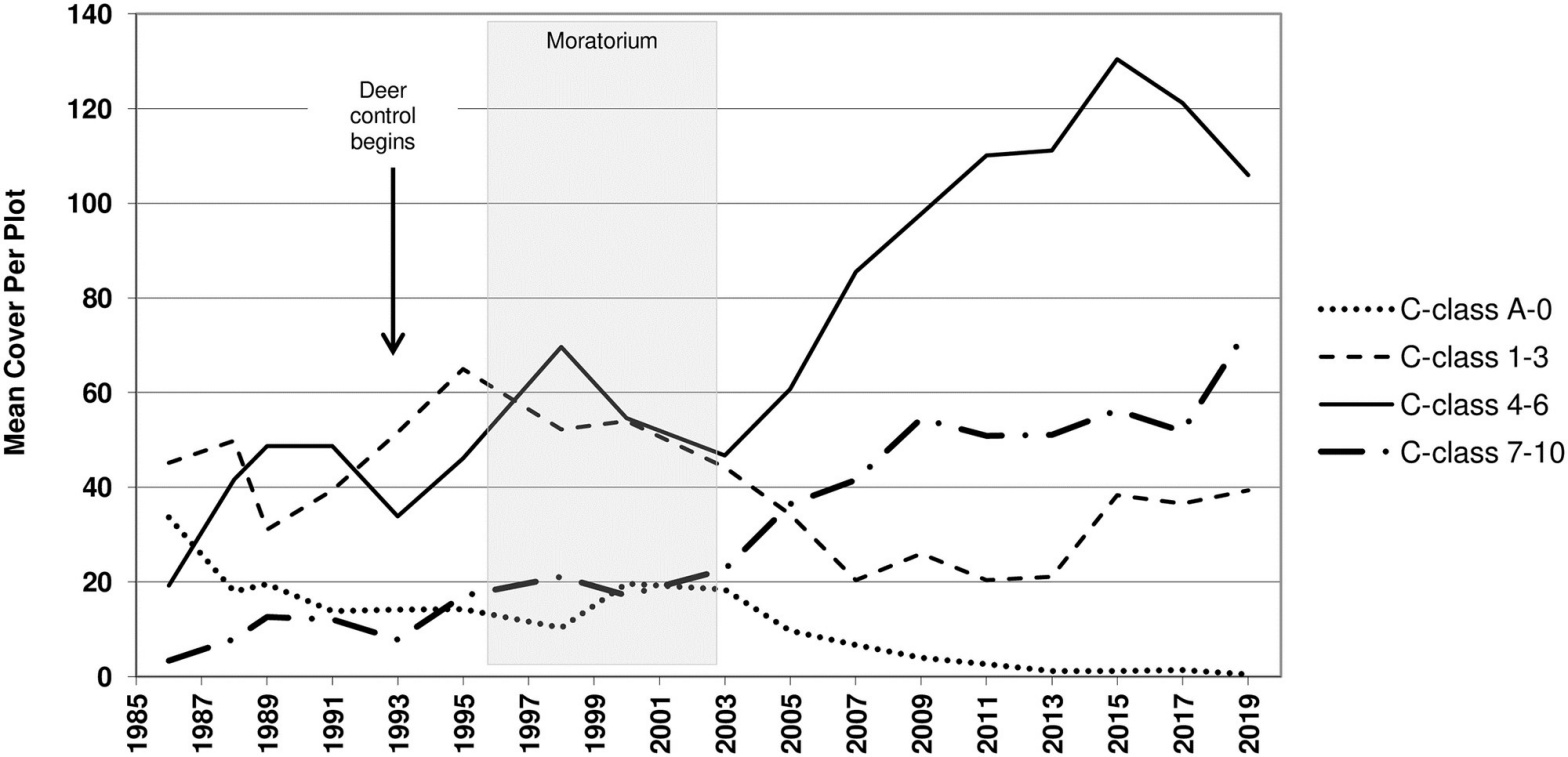
Mean cover per plot of species within each of four conservatism classes. Arrows represent the onset of deer culling, which continued thereafter. Shaded area represents the vegetation management moratorium years.

**Fig 7:**
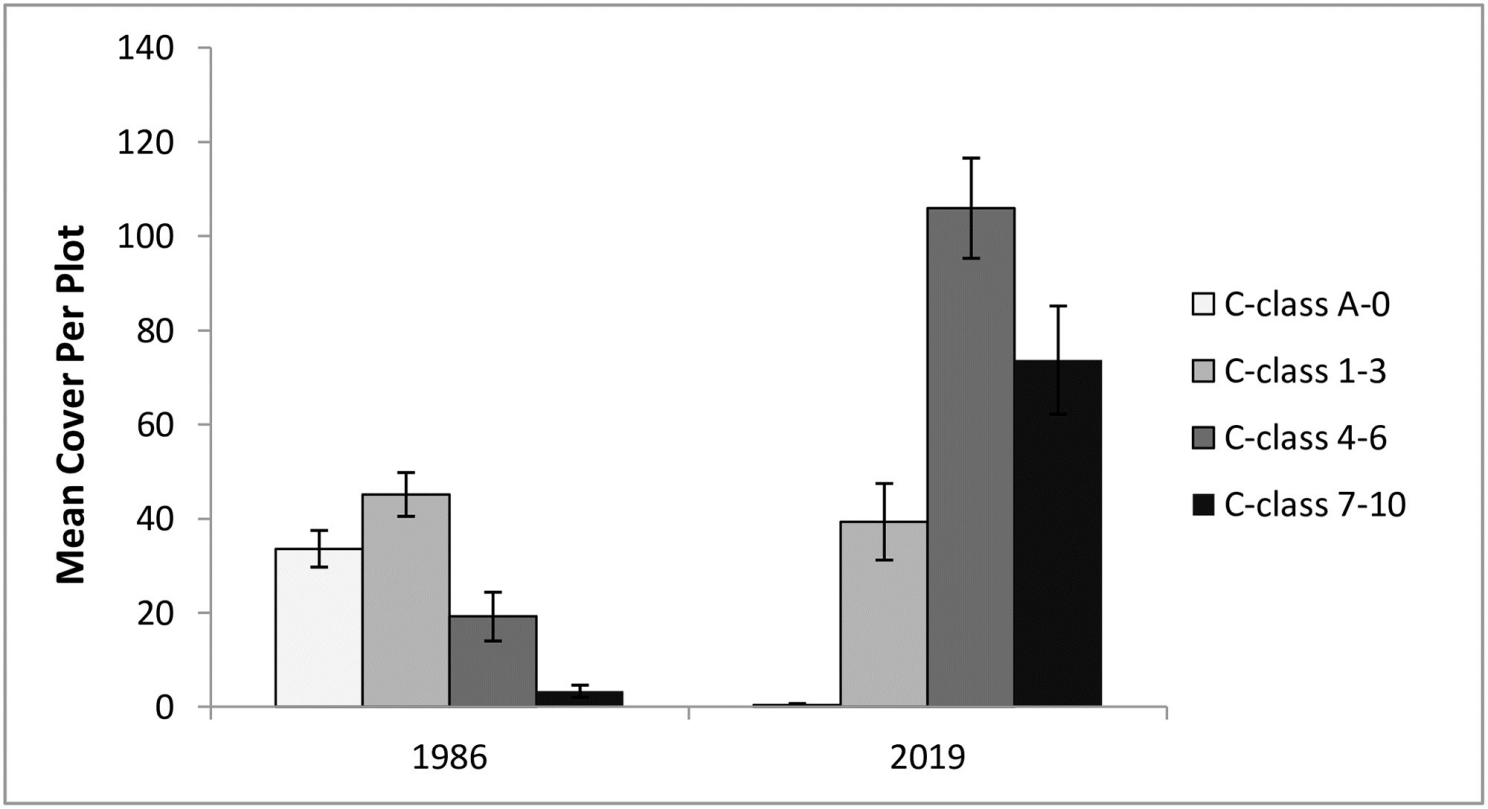
Mean cover per plot of species within each of four conservatism classes in the initial and most recent years of the study. Values are identical to those in Fig 6 but are shown here only for the years included in the repeated measures MANOVA from Table 3. Error bars represent standard errors of the mean.

The abundance of species within each conservatism class showed markedly different qualitative patterns over time (Fig 6), and each conservatism class except C = 1–3 changed significantly from 1986 to 2019 (Table 3; Figs 6 and 7). Finally, regarding non-native species, the 1985–86 plant sample contained 10 non-native species, with cumulative sum cover of 414, while the 2019 sample contained only one, with sum cover of 6 (S2 Table).

**Table 3:** Repeated measures MANOVA comparing cover by species within four conservatism classes for the years 1986 and 2019 (see also Fig 7). Individual ANOVAs for each cover class follow.

Effect	Wilks’ Lambda test statistic	F	Hypothesis *df*	Error *df*	*P*-value
Between subjects (intercept)	0.025	108	4	11	< 0.000 **
Within subjects (time)	0.048	*55*	4	11	< 0.000 **
**ANOVA: C-class A-0**					
Source	*df*	Sum of Squares	Mean Squares	*F-*ratio	*P*-value
Year	1	8267	8267	65.4	< 0.000 **
Residuals	14	1772	127		
**ANOVA: C-class 1–3**					
Source	*df*	Sum of Squares	Mean Squares	*F-*ratio	*P*-value
Year	1	252	252	0.227	0.641
Residuals	14	15,576	1,113		
**ANOVA: C-class 4–6**					
Source	*df*	Sum of Squares	Mean Squares	*F-*ratio	*P*-value
Year	1	56,420	56,420	75.9	< 0.000 **
Residuals	14	10,412	744		
**ANOVA: C-class 7–10**					
Source	*df*	Sum of Squares	Mean Squares	*F-*ratio	*P*-value
Year	1	37,101	37,101	32.2	< 0.000 **
Residuals	14	16,134	1,152		

## Discussion

To summarize the results for each of this study’s objectives: First, we found successful restoration of a degraded oak woodland’s plant community under a long-term, multi-treatment, ecological management regime. Second, of the common metrics studied, Floristic Quality measures most accurately quantified the management success/failure and restoration progress over the life of the study. Third, an abrupt, mid-study cessation of the management treatments stalled or even reversed the woodlands recovery, which then quickly resumed its overall upward progress once management resumed. And, finally, the abundance-weighted Floristic Quality measure, Cover-weighted FQI, was the most sensitive restoration measure: it quickly responded to the mid-study management cessation, which may make it most useful for restoration work that needs to quickly detect and adjust to community changes (i.e., adaptive management).

Restoration and conservation of the biodiversity of oak savannas and woodlands face difficult challenges. As stated by Bowles and McBride [50], “Fire-maintained oak savannas on silt-loam [rich] soils essentially disappeared from midwestern North America soon after European settlement because of fire suppression and agriculture. As a result, there are no precise models for restoring this vegetation, and its management and recovery needs are uncertain.” Though northern Illinois has many small (0.1 to 2-hectare [0.25 to 5-acre]), high-quality prairie remnants on rich soils, dense with conservative plant species, no such savanna/woodland remnants survive [51]. On the other hand, many large, albeit degraded savanna/woodland remnants survive (e.g., 20 to 200-hectare [50 to 500-acre]), and conservation has focused on these areas because they are large and ripe for restoration.

Over the 34-year study period, the vegetation of Vestal Grove has changed from a biologically depauperate woodland dominated by non-native species to a community with Floristic Quality measures essentially undistinguishable from the highest quality remnants of this habitat type. The native Mean *C* rose from 2.3 per plot (2.5 for the site) to 5.0 per plot (4.9 for the site; Fig 2; S2 Table). The initial value is typical of other highly degraded, non-native dominated habitats (e.g., abandoned farm fields, pastures), whereas the final value approaches levels seen in the finest undegraded, remnant natural areas in the region—surpassing what has typically been observed in restorations [31]. Non-native species have functionally been eliminated from the site (see S2 Table).

There is no doubt that a pristine, remnant oak woodland with its full plant assemblage and without modern anthropogenic degradation would have even greater native diversity (α and β) and Floristic Quality numbers than those reached at our study site. This fuller spectrum of species would include a greater diversity of phenologies, specialists, functional groups or guilds [52,53]. But for a reference community comparison, we looked to Middlefork Savanna, a nearby silt-loam oak savanna/woodland complex considered the highest-quality, least degraded, native remnant of this habitat type in the region (i.e., northern Illinois; [51]). Both its mean native richness and native Mean *C* (14.2/plot & 4.1/plot, respectively; [54] and unpublished data) are lower than the values Vestal Grove reached by the end of the study (Figs 1 and 2), confirming what was a very successful restoration.

While other studies have shown strong responses of Floristic Quality measures to ecological management (e.g., deer overgrazing and Mean *C*; [55]), we know of only a handful of instances where a habitat starting from such a degraded state has been so successfully restored (all prairies–[56–58]). Once degraded, habitats are more often difficult to restore, and relatively few projects meet their conservation goals, with FQA and diversity levels peaking at low levels [9,22,59]. We suggest two primary reasons to explain the success documented in the current study. First, most studies have implemented/examined only a single management technique in restoration. Studies investigating more than one technique are less common, but they tend towards greater success (e.g., fire and seeding [60]; fire and stand thinning [61]; and the current study). Second, even fewer studies have monitored management and restoration for as long as the current 34-year study.

Our results strongly suggest that it was the long-term and multi-faceted management regime that explained the remarkable woodland restoration success. The management regime included removal of invasive herbaceous weeds, cutting and herbiciding invasive shrubs and trees, deer control, prescribed fire, and seeding. This study did not test the management-restoration relationship with a spatially replicated, no-management control. However, three strong lines of evidence nonetheless support the treatments as causative. First, unmanaged woods within the same preserve and directly adjacent to the study area showed essentially no change from the highly degraded pre-treatment condition of the study area over the same 34-year period, based on stewards’ observations. These untreated areas have seen none of the return or recovery of the native floras documented in the managed study area. Second, other studies have shown that when left alone, degraded oak forests/woodlands will not recover their previous habitat quality (native diversity and composition of the ground layer flora measured over even centuries), even if they are directly abutted by high-quality, “old-growth” woodland seed sources [1,8]. Finally, the unplanned management moratorium (discussed below) functioned as a temporal control to the study treatments. And, since this no-management period resulted in an immediate, observable dampening in the overall upward trend in conservation measures, and since they quickly rebounded once ecological management resumed (Figs 3 and 4), this unplanned control period provides strong evidence for the causal effects of management. Taken together, these arguments clearly implicate the site’s management regime as the determinant factor in the measured response variables, and, therefore, in the restoration success. It shows that neglect, or relying on time and succession, will not lead to natural area restoration, especially in the highly-degraded, developed landscape characterizing the study region.

Our study did not attempt to isolate contributions from the individual management techniques (fire, seed additions, invasive species control, etc.). Many other studies have examined the impact of single management treatments, and on their own they tend to lead to modest Floristic Quality gains (fire [11,62]; brush removal [12]; soil treatments [22,63]). Therefore, our results suggest that a combination is necessary to achieve restoration outcomes of this magnitude, especially in modern ecosystem contexts. Nonetheless, studies with controlled comparisons of different management techniques are needed to sort out the long-term effects of particular treatments, as well as their interactions, on biodiversity or conservation measures.

We suggest that while each of the treatments employed in this study was crucial to the restoration’s success, the repeated addition of a diverse seed mix was likely the key factor explaining our dramatic results. Of the species recorded in 2019 that were not recorded in the first year of the survey, 82% were included in the seed mixes (S1 and S2 Tables). This suggests that unassisted colonization was not the primary explanation for increased species richness and Floristic Quality.

Native seed addition has been experimentally shown to increase the rate at which both grassland and woodland restorations increase plant biodiversity [23,64,65]. Long-term chronosequences of high-quality prairie restorations show that plot FQIs are best explained by the initial FQI of the restoration seed mix [26]. They also show that seed mix composition strongly influences the restoration’s composition [66]. In our case, the Mean *C* of the seed list was 6.2, compared to a value of 4.9 for the 2019 species list (S1 and S2 Tables). This seeding effect is not surprising given that recovery of plant communities in successional habitats, woodlands, and even mature forests is highly limited by seed [6,67,68]. While woodland studies have most often focused on the lack of recovery by specific groups such as spring ephemerals or slow-dispersing species such as myrmechores (ant-dispersed), we would suggest that conservative species in general should be assumed propagule/seed limited when it comes to habitat restoration. This may be due, but not limited to, their dispersibility (e.g., less seed production, slower growth rates, limited local source populations [69]).

To be sure, even when restorations are generously seeded recovery trends may be erratic and success rates poor—especially when non-native species invade [9]. It is difficult to compare these results to similar efforts, because few studies have been published that combine use of repeated seed additions over several years with a full range of management treatments (but see [23]).

### Differences in conservation and biodiversity indices

The null hypotheses for the models tested were that of no predictable change over time. For most FQA measures there was strong statistical support for an increasing overall trend. However, Cover-weighted FQI models showed weak statistical justification for rejecting the null (despite what appeared to be a dramatic visual increase in this metric over time; Fig 4). We suggest that the lack of statistical support for change over time was caused by deviation to the otherwise increasing trend line in the middle of the study period. This “noise” within the long-term pattern affected the model’s fit and statistical significance and implicates an exogenous factor that outweighed the effects of time alone on the trend. That the restoration’s increasing trend line was interrupted by deer overpopulation and the management moratorium is actually an accurate representation of the ecology of the site. In other words, the weak model fit should have actually been expected, if the metric was accurately reflecting a temporarily stalled recovery due to the externally imposed disruption in ecological management. This is compared to metrics like FQI and Mean *C* that showed a strong overall trend according to the test statistic, because their trend lines showed less dramatic mid-study perturbations during the management moratorium (Figs 2 and 3). In this way, the Cover-weighted FQI metric was quicker to respond and more sensitive to acute vegetation changes.

This ability to detect ecosystem degradation or changes in restoration trajectory sooner than other ecological metrics could be crucial to land managers’ ‘real-time’ decision making. For example, Cover-weighted FQI’s apparent reflection of overabundant deer or excessive invasive species could warn land managers of reductions in the size or robustness of browsed or shaded species before their populations are eliminated from a site. However, there are also reasons why abundance-weighted measures may be less appropriate for long-term assessments of the Floristic Quality of restoration sites, as discussed by Wilhelm and Ladd [70]. They caution that species with inherently larger growth forms or high population densities could unduly influence abundance-weighted measures. Conversely, other authors question whether such measures de-value species that are inherently rare in plant communities [71]. Some studies of abundance-weighted FQA measures have found little or no improvement over non-weighted measures [72,73] or improvement only in a small subset of habitats [74]. Other studies have found dramatic responses of abundance-weighted FQA metrics to invasive species control, burning, and deer control [55,75]. More research is needed to determine the circumstances or purposes for which abundance-weighted FQA measures are more useful than non-weighted measures.

Species richness and evenness provide intuitive, relevant measures that can be easily understood and communicated. Many studies have relied solely on species richness or evenness as a measure of diversity (e.g., [28,29,76,77]), and they can be somewhat responsive to management (fire [17,78]; deer control [55]; fire and thinning [79]), or relatively unaffected by them (deer control [80]).

In our study, species richness showed no significant change over time—it reached its overall mean value early in the study and remained stable thereafter. Despite the increase in species richness, to have relied on this index alone to describe this site would have led to the incorrect conclusion that little had changed since 1988. Mean H’ (Shannon-Weiner Index) or evenness also showed no overall change over time and would have supported this incorrect conclusion. On their own, these measures would have been inadequate for making conservation decisions at the study site, compared to indices that more directly incorporated changes in species composition. Taft et al. [60] also found Mean *C* and FQI to be better than several standard diversity indices (e.g. species richness, Simpson Index, Shannon-Weiner Index) for distinguishing between relatively disturbed remnant and reconstructed prairie units (but see [52]).

The long-term increase we observed in Floristic Quality, but not necessarily diversity, is perhaps best deconstructed using the data shown in Figs 6 and 7 and S2 Table, which provide a detailed portrait of community compositional change. Here, we illustrate species turnover from a badly degraded community toward one comprised of a diverse array of species types that included those of high-quality, remnant habitats. These most conservative species (C = 7–10) did not appear to change rapidly during the management disruption, but instead steadily increased over time, perhaps in response to the establishment of a native species matrix and the return of natural processes such as fire. The least conservative species (C = A-0) declined steadily, with the decline slowed during periods of management neglect. It was, however, the species of intermediate C-value that fluctuated more widely and were perhaps more responsive to the two periods of management disruption. We suggest that separating species into classes this way and observing their distinct changes provides a nuanced perspective that will allow restoration practitioners to more fully understand and respond to changes in the plant community.

## Conclusions

This study adds to the growing body of work demonstrating the effectiveness of ecological management of oak woodlands that includes regular fire, control of invasive species and overabundant deer, and repeated additions of a diverse seed mix. Our study underscores the importance of continued, long-term management to achieve restorations that approach remnant quality, and it illustrates the speed with which vegetation communities can respond (negatively) to neglect and (positively) to management. This study found that per-plot, Cover-weighted FQI, which incorporates species richness, abundance, and conservatism, may be a particularly useful and versatile metric to describe both short-term and long-term changes in plant communities. Breaking the data into conservatism classes helped us detail and further understand the compositional changes in the plant community over time.

Our experience during the moratorium years reinforces the lesson that elected officials, administrators, the media, and the public must be given access to readily understandable measures of ecosystem health or natural area value if they are to understand and support decisions based on science and best management practices. Quantitative analyses like these can provide clear explanations of why tax money is being spent on sometimes counter-intuitive restoration management techniques, and how these techniques are effective. Integrated biodiversity and conservation measures like Floristic Quality Assessment can be especially helpful because they are both easily understood and scientifically validated.

## Supporting information

S1 Table Species seeded into Vestal Grove. “C” represents the species’ Coefficient of Conservatism, using values from [31]. Species nomenclature has been updated to that of [81].
(PDF)

S2 Table Species lists for the initial and most recent sample years. “Sum Cover” represents the summed cover for each species over all 15 plots for that year. “C” represents the species’ Coefficient of Conservatism, using values from [31]. Species nomenclature has been updated to that of [81]. Species in bold are adventive. Species marked with “*” in the 2019 list were *not* found in the 1985–6 sample and *were* present in the seed mix (see S1 Table).
(PDF)
