# Supplementary material for: Dramatic long-term restoration of an oak woodland due to multiple, sustained management treatments
Source: PLoS One. 2020 Oct 23;15(10):e0241061. doi: 10.1371/journal.pone.0241061 (PMC7584219; doi:10.1371/journal.pone.0241061)
Supplement: S1 Table — “C” represents the species’ Coefficient of Conservatism, using values from [31]. Species nomenclature has been updated to that of [81]. (PDF) [file pone.0241061.s001.pdf]

| Species seeded into Vestal Grove | *C* |
|---|---|
| *Actaea pachypoda* | 7 |
| *Agastache nepetoides* | 5 |
| *Agastache scrophulariaefolia* | 5 |
| *Ageratina altissima* | 4 |
| *Agrostis perennans* | 3 |
| *Allium cernuum* | 7 |
| *Amphicarpaea bracteata* | 4 |
| *Anemone virginiana* | 5 |
| *Anemonella thalictroides* | 7 |
| *Aralia racemosa* | 10 |
| *Arisaema dracontium* | 7 |
| *Arisaema triphyllum* | 4 |
| *Arnoglossum atriplicifolium* | 8 |
| *Asclepias exaltata* | 9 |
| *Aureolaria grandiflora pulchra* | 8 |
| *Blephilia hirsuta* | 8 |
| *Boechera laevigata* | 5 |
| *Brachyelytrum erectum* | 10 |
| *Bromus latiglumis* | 5 |
| *Bromus nottowayanus* | 5 |
| *Camassia scilloides* | 6 |
| *Campanulastrum americanum* | 3 |

| | |
|---|---|
| *Carex cephalophora* | 3 |
| *Carex cristatella* | 4 |
| *Carex davisii* | 7 |
| *Carex formosa* | 10 |
| *Carex gracillima* | 10 |
| *Carex granularis* | 4 |
| *Carex grayi* | 7 |
| *Carex hirsutella* | 4 |
| *Carex pensylvanica* | 5 |
| *Carex rosea* | 4 |
| *Carex shortiana* | 10 |
| *Carex sprengelii* | 9 |
| *Carex swanii* | 8 |
| *Caulophyllum thalictroides* | 8 |
| *Cinna arundinacea* | 5 |
| *Cirsium altissimum* | 6 |
| *Claytonia virginica* | 2 |
| *Conopholis americana* | 10 |
| *Coreopsis tripteris* | 5 |
| *Danthonia spicata* | 3 |
| *Dasistoma macrophylla* | 8 |
| *Dentaria laciniata* | 5 |
| *Desmodium cuspidatum* | 8 |

| | |
|---|---|
| *Desmodium glutinosum* | 5 |
| *Dioscorea villosa* | 7 |
| *Dodecatheon meadia* | 6 |
| *Elymus hystrix* | 5 |
| *Elymus villosus* | 5 |
| *Elymus virginicus* | 4 |
| *Erythronium albidum* | 5 |
| *Euonymus atropurpureus* | 8 |
| *Euonymus obovatus* | 7 |
| *Eurybia macrophylla* | 8 |
| *Eutrochium purpureum* | 7 |
| *Festuca subverticillata* | 5 |
| *Floerkea proserpinacoides* | 7 |
| *Galium circaezans* | 7 |
| *Gentiana alba* | 9 |
| *Glyceria striata* | 4 |
| *Hamamelis virginiana* | 8 |
| *Hedeoma pulegiodes* | 3 |
| *Heliopsis helianthoides* | 5 |
| *Heracleum maximum* | 5 |
| *Hypericum ascyron* | 10 |
| *Hypericum prolificum* | 9 |
| *Iodanthus pinnatifidus* | 8 |

| | |
|---|---|
| *Lactuca biennis* | 4 |
| *Lactuca canadensis* | 2 |
| *Lactuca floridana* | 5 |
| *Leersia virginica* | 7 |
| *Lespedeza frutescens* | 7 |
| *Lithospermum latifolium* | 9 |
| *Lobelia siphilitica* | 6 |
| *Lonicera reticulata* | 7 |
| *Menispermum canadense* | 6 |
| *Muhlenbergia mexicana* | 5 |
| *Osmorhiza claytonii* | 3 |
| *Ostrya virginiana* | 5 |
| *Pedicularis canadensis* | 9 |
| *Penstemon digitalis* | 7 |
| *Perideridia americana* | 8 |
| *Phlox divaricata* | 5 |
| *Phryma leptostachya* | 4 |
| *Polemonium reptans* | 5 |
| *Polygonatum biflorum* | 3 |
| *Nabalus alba* | 5 |
| *Nabalus altissima* | 8 |
| *Ribes americanum* | 7 |
| *Roegneria subsecunda* | 8 |

| | |
|---|---|
| *Rosa blanda* | 5 |
| *Rosa setigera* | 7 |
| *Rudbeckia subtomentosa* | 9 |
| *Rudbeckia triloba* | 3 |
| *Sanguinaria canadensis* | 6 |
| *Sanicula marilandica* | 6 |
| *Sanicula odorata* | 2 |
| *Scrophularia marilandica* | 4 |
| *Silene stellata* | 6 |
| *Sisyrinchium angustifolium* | 10 |
| *Smilacina racemosa* | 3 |
| *Smilacina stellata* | 5 |
| *Smilax ecirrhata* | 5 |
| *Smilax lasioneuron* | 5 |
| *Smilax tamnoides hispida* | 5 |
| *Solidago caesia* | 7 |
| *Solidago flexicaulis* | 7 |
| *Solidago speciosa* | 7 |
| *Solidago ulmifolia* | 5 |
| *Sphenopholis intermedia* | 4 |
| *Staphylea trifolia* | 7 |
| *Symphyotrichum lateriflorum* | 4 |
| *Symphyotrichum oolentangiense* | 8 |

| | |
|---|---|
| *Symphyotrichum shortii* | 8 |
| *Taenidia integerrima* | 9 |
| *Thalictrum dioicum* | 7 |
| *Thalictrum thalictroides* | 7 |
| *Thaspium trifoliatum* | 7 |
| *Tradescantia ohiensis* | 2 |
| *Trillium grandiflorum* | 8 |
| *Trillium recurvatum* | 5 |
| *Triosteum aurantiacum* | 5 |
| *Triosteum perfoliatum* | 5 |
| *Uvularia grandiflora* | 7 |
| *Viburnum acerifolium* | 9 |
| *Viola labradorica* | 6 |
| *Viola pubescens* | 5 |
| *Zizia aurea* | 7 |
| Seed mix Mean *C* | 6.2 |
