# Supplementary material for: Dramatic long-term restoration of an oak woodland due to multiple, sustained management treatments
Source: PLoS One. 2020 Oct 23;15(10):e0241061. doi: 10.1371/journal.pone.0241061 (PMC7584219; doi:10.1371/journal.pone.0241061)
Supplement: S2 Table — “Sum Cover” represents the summed cover for each species over all 15 plots for that year. “C” represents the species’ Coefficient of Conservatism, using values from [31]. Species nomenclature has been updated to that of [81]. Species in bold are adventive. Species marked with “*” in the 2019 list were not found in the 1985–6 sample and were present in the seed mix (see S1 Table). (PDF) [file pone.0241061.s002.pdf]

1985-6

| Species | Sum Cover | *C* |
|---|---|---|
| ***Rhamnus cathartica*** | 261 | 0 |
| *Circaea canadensis* | 95 | 1 |
| *Geum canadense* | 86 | 1 |
| *Arisaema triphyllum* | 80 | 4 |
| *Cornus racemosa* | 73 | 1 |
| *Vitis riparia* | 68 | 2 |
| ***Solanum dulcamara*** | 63 | 0 |
| *Prunella vulgaris* ssp. *lanceolata* | 55 | 0 |
| *Quercus macrocarpa* | 50 | 5 |
| *Rubus occidentalis* | 46 | 2 |
| *Geranium maculatum* | 45 | 4 |
| *Epilobium coloratum* | 40 | 3 |
| *Prunus virginiana* | 40 | 3 |
| *Smilacina racemosa* | 40 | 3 |
| *Oxalis stricta* | 35 | 0 |
| *Ranunculus septentrionalis* | 35 | 5 |
| *Toxicodendron radicans* | 33 | 2 |
| *Potentilla simplex* | 30 | 4 |
| ***Lonicera tatarica*** | 25 | 0 |
| *Parthenocissus inserta* | 25 | 1 |

2019

| Species | Sum Cover | *C* |
|---|---|---|
| *Helianthus strumosus* | 352 | 5 |
| *Floerkea proserpinacoides** | 228 | 7 |
| *Ranunculus septentrionalis* | 197 | 5 |
| *Solidago altissima* | 192 | 1 |
| *Zizia aurea** | 190 | 7 |
| *Solidago ulmifolia** | 180 | 5 |
| *Amphicarpaea bracteata** | 155 | 4 |
| *Leersia virginica** | 135 | 7 |
| *Eutrochium purpureum** | 130 | 7 |
| *Cryptotaenia canadensis* | 109 | 2 |
| *Geranium maculatum* | 104 | 4 |
| *Arisaema triphyllum* | 74 | 4 |
| *Brachyelytrum erectum** | 66 | 10 |
| *Camassia scilloides** | 56 | 6 |
| *Rudbeckia subtomentosa** | 52 | 9 |
| *Pedicularis canadensis** | 50 | 9 |
| *Heracleum maximum** | 49 | 5 |
| *Lithospermum latifolium** | 46 | 9 |
| *Triosteum perfoliatum** | 45 | 5 |
| *Carex davisii** | 40 | 7 |

| | | |
|---|---|---|
| ***Taraxacum officinale*** | 25 | 0 |
| *Allium canadense* | 20 | 2 |
| *Allium cernuum* | 20 | 7 |
| *Allium tricoccum* | 20 | 6 |
| *Prunus serotina* | 20 | 1 |
| *Carex blanda* | 18 | 1 |
| *Aquilegia canadensis* | 15 | 6 |
| *Crataegus mollis* | 15 | 2 |
| *Cirsium discolor* | 10 | 2 |
| *Fragaria virginiana* | 10 | 1 |
| ***Poa pratensis*** | 10 | 0 |
| *Populus deltoides* | 10 | 2 |
| *Rubus idaeus* ssp. *strigosus* | 10 | 3 |
| *Viola sororia* | 10 | 3 |
| *Agrimonia gryposepala* | 5 | 2 |
| ***Arctium minus*** | 5 | 0 |
| ***Cirsium vulgare*** | 5 | 0 |
| ***Epipactis helleborine*** | 5 | 0 |
| *Erigeron annuus* | 5 | 0 |
| *Nabalus albus* | 5 | 5 |
| ***Frangula alnus*** | 5 | 0 |
| *Ribes americanum* | 5 | 7 |
| *Smilax ecirrhata* | 5 | 5 |

| | | |
|---|---|---|
| *Dentaria laciniata** | 40 | 5 |
| *Rubus occidentalis* | 40 | 2 |
| *Thalictrum dioicum** | 38 | 7 |
| *Symphyotrichum shortii** | 35 | 8 |
| *Nabalus albus* | 34 | 5 |
| *Carex blanda* | 33 | 1 |
| *Smilax lasioneuron* | 33 | 5 |
| *Sanguinaria canadensis** | 30 | 6 |
| *Viola pubescens** | 30 | 5 |
| *Allium canadense* | 28 | 2 |
| *Claytonia virginica** | 28 | 2 |
| *Polygonatum biflorum** | 27 | 3 |
| *Carex pensylvanica** | 26 | 5 |
| *Galium aparine* | 25 | 1 |
| *Sanicula odorata** | 25 | 2 |
| *Allium tricoccum* | 23 | 7 |
| *Cinna arundinacea** | 23 | 5 |
| *Allium burdickii* | 20 | 6 |
| *Penstemon digitalis** | 20 | 4 |
| *Perideridia americana** | 20 | 8 |
| *Silene stellata** | 20 | 6 |
| *Carex cephalophora** | 18 | 3 |
| *Impatiens capensis* | 18 | 3 |

| | | |
|---|---|---|
| *Smilax lasioneuron* | 5 | 5 |
| *Solidago juncea* | 5 | 5 |
| ***Sonchus asper*** | 5 | 0 |
| *Verbena hastata* | 5 | 4 |
| ***Viburnum opulus*** | 5 | 0 |
| *Symphyotrichum drummondii* | 3 | 2 |
| *Populus tremuloides* | 3 | 4 |
| | | |
| | | |
| | | |
| | | |
| | | |
| | | |
| | | |
| | | |
| | | |
| | | |
| | | |
| | | |
| | | |
| | | |

| | | |
|---|---|---|
| *Trillium recurvatum** | 18 | 5 |
| *Elymus hystrix** | 13 | 5 |
| *Smilax ecirrhata* | 12 | 5 |
| *Persicaria virginiana* | 10 | 2 |
| *Solidago caesia** | 10 | 7 |
| *Thalictrum revolutum* | 10 | 6 |
| *Erythronium albidum** | 9 | 5 |
| *Symphyotrichum lateriflorum** | 8 | 4 |
| *Cirsium altissimum** | 8 | 6 |
| *Elymus virginicus** | 8 | 4 |
| *Fragaria virginiana* | 8 | 1 |
| *Rubus flagellaris* | 8 | 3 |
| *Smilacina racemosa* | 8 | 3 |
| *Allium cernuum* | 7 | 7 |
| *Potentilla simplex* | 6 | 4 |
| ***Taraxacum officinale*** | 6 | 0 |
| *Trillium grandiflorum** | 6 | 8 |
| *Eurybia macrophylla** | 5 | 8 |
| *Bromus latiglumis** | 5 | 5 |
| *Carex rosea** | 5 | 4 |
| *Ageratina altissima** | 5 | 4 |
| *Polemonium reptans** | 5 | 5 |

| | | |
|---|---|---|
| | | |
| | | |
| | | |
| | | |
| | | |
| | | |
| | | |
| | | |
| Species list Mean *C* | | 2.5 |

| | | |
|---|---|---|
| *Smilacina stellata** | 5 | 5 |
| *Tradescantia ohiensis** | 5 | 2 |
| *Vitis riparia* | 5 | 2 |
| *Arisaema dracontium** | 3 | 7 |
| *Bromus nottowayanus** | 3 | 5 |
| *Geum canadense* | 3 | 1 |
| *Thalictrum thalictroides** | 1 | 7 |
| *Carex radiata* | 1 | 6 |
| Species list Mean *C* | | 4.9 |
